# Needs of the mind: how aptic normativity can guide conceptual adaptation

**DOI:** 10.1007/s11098-026-02511-3

**Published:** 2026-03-14

**Authors:** Matthieu Queloz

**Affiliations:** Ambizione Research Fellow of the Swiss National Science Foundation, Institute of Philosophy, University of Bern, Laenggassstrasse 49a, 3012 Bern, Switzerland

**Keywords:** Cognitive needs, Normativity, Fittingness, Concepts, Hermeneutical injustice, Conceptual adaptation, Conceptual ethics, Conceptual engineering, Privacy

## Abstract

How to appraise concepts? Against approaches focusing either on the goals of concept-users or on the functions of concepts, I advocate focusing on what concepts we now *need*. After diagnosing the historical ambivalence of “need” between subjective want and objective exigency, I characterise conceptual needs as possessing a distinctively *aptic* normativity – a normativity of fittingness. They signal a cognitive privation that marks a mismatch between our conceptual repertoire and our situation, reorienting conceptual engineering from detached *amelioration* to situated *adaptation*. To render this aptic normativity tractable, I introduce the analytic device of “need matrices.” As illustrated using the concept of *privacy*, need matrices and the “need vectors” they generate provide blueprints for fitting concepts by modelling needs as arising at the intersection of concerns, capacities, and circumstances. Unlike function-first approaches, the need-first approach is inherently prospective rather than retrospective; unlike goal-first approaches, it acknowledges that concepts are answerable to pressures we face unwittingly and unwillingly. Starting from potentially unprecedented predicaments rather than established goals or functions makes this approach uniquely suited to guiding conceptual adaptation in times of social and technological upheaval.

## Conceptual needs and conceptual adaptation

The meteoric rise of conceptual ethics and conceptual engineering, which seek to assess and ameliorate our conceptual resources, has sharpened a basic question: by what standard should we appraise concepts? Two prominent answers look either to (i) the *goals* (or aims or purposes) of concept-users or to (ii) the *functions* of concepts. I argue that both approaches miss an important notion that our ordinary language already marks: the notion of *need*. My aim is to propose an approach on which the fittingness of our concepts to our evolving needs – rather than their intended goals or inherited functions – provides the primary standard of appraisal, and to show that the forward-looking notion of need involved in this appraisal is particularly well-suited to guiding conceptual adaptation.^1^

In contemporary philosophy, needs get sustained attention mainly in discussions of the special moral and political claims arising from basic bodily needs.^2^ But there are also *needs of the mind*. I do not mean emotional needs (such as the need to feel accepted), but distinctively *cognitive* needs. These are undertheorised and typically overshadowed by physiological and psychological needs. Abraham Maslow’s well-known “hierarchy of needs,” for example, originally centred on physiological needs (air, food, and shelter) and psychological needs (love, self-esteem, and self-actualisation). Only later did Maslow add “cognitive needs” – but construed so broadly, as generic needs for knowledge, understanding, and meaning, that they failed to register the species of cognitive need that matters most for conceptual ethics and engineering: our *conceptual* needs, which is to say our needs for specific concepts or conceptions. Our minds can be needful simply in virtue of lacking certain concepts.

In the context of conceptual engineering, the very notion of “conceptual needs” invites a shift in focus. Whereas many conceptual engineering projects – from Carnap (1962) to Scharp (2013, 2020) and Cappelen (2018, 2020) – aspire to ameliorate our existing concepts by fixing perceived defects inherent in them, such as vagueness or inconsistency, framing the issue in terms of conceptual needs reminds us that our conceptual repertoire can fall short not because our existing concepts are inherently flawed, but because we lack the particular concepts required to navigate novel circumstances.

Instead of calling for conceptual *amelioration*, this calls for conceptual *adaptation*: the process of adjusting our conceptual repertoire to keep step with our evolving conceptual needs. Where conceptual amelioration channelled attention towards inherent deficiencies of concepts, framing the issue in terms of conceptual needs directs our attention outwards, to the relation between our conceptual repertoire and our circumstances. Conceptual needs arise from mismatches between these two relata. When circumstances change, concepts that once served us well may cease to meet our needs – not because they have become defective, but because our situation has evolved. This is why, as Jeroen Hopster and Guido Löhr (2023) have compellingly argued, the aspirations of conceptual engineering should include conceptual adaptation alongside amelioration.

While Hopster and Löhr conceive of adaptation primarily as preserving a concept’s function amid changing circumstances, however, I submit that conceptual adaptation is better understood as adjusting our conceptual repertoire to meet our evolving conceptual needs. Such a *need-first* approach suggests a more forward-looking role for conceptual engineering. It is not necessarily about preserving established functions, but encompasses adaptation to new needs. This *might* involve finding ways to preserve established functions into novel contexts; but equally, it might require going against established functions or fulfilling entirely new functions. When circumstances are unprecedented, our most pressing conceptual needs may be for concepts performing functions hitherto never discharged by concepts because they never *needed* to be discharged. Even if we agree with J. L. Austin (1961, 133; 1962, 63) that the ingenuity of our inherited conceptual repertoire, honed over generations, surpasses the ingenuity of any individual conceptual engineer, lacking concepts need not be a matter of lacking the conceptual means to extend old functions into new contexts. Truly novel challenges might well call for equally novel solutions. Indeed, even old functions must have been new functions at some point; as Bernard Williams once wrote in response to Austin: “In language, as in politics, the conservative runs into the fact that the old is only what used to be new” (2014, 44). Guidance by established functions alone cannot be enough for effective conceptual adaptation. It must be responsive to what our conceptual needs *now* are.

A parallel argument applies once we consider *whose* conceptual needs are at issue. Those who benefit from inherited functions may well need them to be preserved; but those at whose expense those functions are being fulfilled need them *not* to be preserved; and once this fact dawns on them, they will want to adapt their conceptual repertoire to this realisation about what their distinctive conceptual needs are.

Conceptual needs should not be confused with another, more familiar kind of lack, namely the lack of words. Lacking concepts is importantly different from lacking words. When Max Frisch’s Stiller exclaims: “I lack the language to describe my reality” (Frisch, 1954, p. 84), he registers his struggle to *verbalise* his experience. Martin Kusch has referred to this type of situation as *Sprachnot* or “linguistic despair” (2017).^3^ Yet this describes a mind’s struggle to verbalise a reality or experience that is already intelligible to it – it manifests in despair because it is *all too* intelligible.

By contrast, when we lack not just words, but the very concepts that structure that experience, we are tone-deaf to certain tonalities of reality: we are incapable even of *experiencing* those aspects of it to begin with. Where concepts are lacking, our minds are imperceptibly yet firmly barred from making sense of things in certain ways. A lack of concepts operates “behind our backs, out of our sight, since it limits what we are so much as capable of being aware of” (Brandom, 2001, p. 78). Goethe’s Mephistopheles may seem to deny this when he quips: “*wo Begriffe fehlen*,* da stellt ein Wort zur rechten Zeit sich ein*” – where concepts are lacking, a word conveniently steps in. But the cynicism is the point: words are no substitute for understanding; they merely paper over the gap without closing it. The lack of concepts is a deeper privation than the lack of words.^4^

Unlike a lack of words, a lack of concepts also typically manifests not in despair, but in *ignorance*. While linguistic despair is essentially something one experiences, being conceptually ignorant of aspects of reality is a condition that precisely precludes its own experience.

Occasionally, conceptual ignorance is a blessing. Ruth Millikan reflects in her Dewey Lecture that she only made it through Yale in the 1960s because she lacked the concept of sexism:

What you have no idea could exist you don’t see, or at least you don’t understand … My innocence was surely a very great blessing. Had I been aware of some of the attitudes present then, of things I thought back about or that other people told me about later, I doubt that I would have come through. (Millikan, 2012, p. 9)

Yet few of us would want to unsee what we come to see by acquiring a concept. A conceptual lack is a genuine privation: not just because it blocks one from entertaining certain thoughts and possibilities, but also because it forecloses the individual and social benefits of organising our affairs in terms of a concept. As the concept of sexism illustrates, this can be a matter of reorganising our affairs to *avoid* what the concept refers to. But it can also, more positively, be a matter of building up and stabilising arrangements and institutions enabled by the way of seeing the world that a concept introduces. Hume’s classic discussion of the concept of property is a case in point: acquiring that concept does not merely unlock new thoughts; it furnishes a powerful – if double-edged – tool for social coexistence.^5^ By gaining the ability to conceptualise things as *mine* or *yours*, we gain a resource for reducing conflict over external goods by stabilising their distribution – though, as Rousseau’s account of the origins of inequality warned, not without cost.^6^

Conceptual needs, which turn a mere *absence* of concepts into a *lack*, are therefore crucial for conceptual engineering because they point beyond our current repertoire, indicating where new ways of thinking are likely to be needed and what these would have to be like. Although we cannot think with concepts we do not yet have, we *can* become aware of our needs for such concepts in advance of having them. The needs of the mind then serve as a compass, orienting us toward concepts we do not yet possess, but already need. And by understanding what exactly the need is a need *for*, we gain a blueprint for the kind of concept that would meet it.

But what exactly *are* conceptual needs? How do they differ from other kinds of needs? And what differentiates concept appraisal in terms of conceptual needs from appraisals based on goals or conceptual functions?

To answer these questions, I proceed as follows: § 2 traces the genealogy of “need” to diagnose the notion’s present oscillation between objective exigency and subjective want. § 3 argues that conceptual needs have a distinctively *aptic* normativity: they register fittingness, not deontic requirement or evaluative goodness. § 4 lays out and illustrates a need-first approach to conceptual adaptation. § 5 argues that this need-first approach offers a less intellectualist alternative to goal-first approaches and a more prospective alternative to function-first approaches.

## The ambivalence of need: a genealogy

If the concept of need is to serve as a standard for conceptual adaptation, we must confront a difficulty: in both ordinary and philosophical usage, the notion seems to oscillate between objective necessity and subjective yearning; sometimes, it marks what must obtain if an organism or an institution is to be viable at all; sometimes, it marks a contingent, psychological sense of lack. Talk of “needs of the mind” inherits this ambiguity. This poses a problem for conceptual engineering: such talk can be heard either as gesturing towards hard constraints on any adequate way of thinking, or, at the other extreme, as a rhetorical elevation of mere wants. As neoclassical economists like to say:^7^ “There are no such things as human ‘needs’ … There are only human wants” (Jensen & Meckling, 1994, p. 7).

We can understand why the notion of need carries these ambivalent resonances by briefly reconstructing its philosophical history. This will bring out how needs have been cast, in different periods, as demands of nature, sentiments of the subject, products of social organisation, and objects of scientific measurement. Prying open this conceptual space will in turn help us situate “conceptual needs” in that space.

The phrase “needs of the mind” itself hails from Denis Diderot’s entry on “*besoin*” (need) in the *Encyclopédie*. In that canonical statement of Enlightenment thought, Diderot defines a “need” in general as “a disagreeable sentiment occasioned by the perceived absence and the desired presence of an object.” He then distinguishes “needs of the body” (*besoins du corps*) from “needs of the mind” (*besoins de l’esprit*). A need of the mind is not something one possesses in virtue of one’s nature or destiny, on this view, but something one *experiences*: it is the experience of lacking something one desires.

Diderot’s conception of needs of the mind marks a sharp break from ancient and medieval conceptions of need, which tended to emphasise how needs could take the form of objective constraints – exigencies imposed by nature, fate, or divine preordination.^8^ These older conceptions of needs as imposed from without are strikingly absent from the *Encyclopédie* entry. Instead, Diderot, in line with the broader Enlightenment tendency to interiorise formerly external norms and necessities, *subjectivises* needs, conceiving of them entirely in psychological terms, as *essentially felt* lacks springing ultimately from our bodily appetites and desires.

This subjectivisation was radicalised by Diderot’s contemporary, Jean-Jacques Rousseau, who distinguished “natural needs” from “artificial needs” (1977). For Rousseau, needs are not merely *felt*, but *made*. In addition to satisfying pre-existing needs, society creates new needs (e.g., for status, luxury, and esteem) that become sources of dependence.

By conceptualising needs as a product of social arrangements, Rousseau laid the groundwork for a more thorough *historicisation* of needs in the nineteenth century. Thinkers such as G. W. F. Hegel saw the “system of needs” (1991, III.2) not as a static feature of the human condition, but as something that evolved with the development of civil society and the state. Needs were produced and refined through social and historical progress. Karl Marx gave this a materialist edge by arguing that it is new modes of production that create new needs and turn what were once luxuries into perceived necessities (Chitty, 1993). This resonates with the way the advertising industry has since exacerbated this tendency by rhetorically inflating “wants” into “needs.”

Yet the nineteenth century also harboured a countercurrent to this increasing subjectivisation of need. The post-Revolutionary era saw the rise of a new scientific objectivism, which expressed itself notably in attempts to ground the social order in precise measurements of need. As Dana Simmons (2015) documents, this new “science of human needs” sought to put the modern wage economy and the welfare state on a scientific basis by quantifying *sociohistorically specific* physical and social needs. Using emerging technologies of measurement such as calorimeters, demographic surveys, and statistics, the concept of a “vital minimum” was articulated as a *dynamic* measure of needs. Needs were no longer a timeless fact of nature. But they were not subjective either; they were the objective and quantifiable product of historical and social conditions. Contemporary attempts to ground an increasing number of human rights in human needs are heirs to this objectivising countertrend (de Neufville, 1982; Moyn, 2018, ch. 5).

This brief historical sketch – from ancient objectivity through Enlightenment subjectivity to the dual nineteenth-century projects of historicism and scientific measurement – helps situate my account in relation to the different resonances that the notion of need carries. In how we think and talk about needs today, objective and subjective aspects coexist. Our potted genealogy of the concept of need suggests that this ambivalence is not the artefact of a wobbly grip on the concept, but is really there in the concept itself, as the sediment of its history. Indeed, the German language explicitly marks this ambivalence by retaining two closely related words for need, *Bedarf* and *Bedürfnis*. *Bedarf* typically refers to an objective requirement or structural demand, which is there independently of whether it is felt. *Bedürfnis*, by contrast, points to a subjective, felt need – a desire or longing that registers as a lack.

These twin words encapsulate two poles of a spectrum, helpfully structuring the space in which I want to carve out a conception of conceptual needs capable of guiding conceptual adaptation. One can talk about what is needed by reference to external standards of adequacy (*Bedarf*), or by reference to an agent’s experience of deprivation (*Bedürfnis*).

Now, for the purposes of conceptual engineering, it is clear that the Enlightenment tendency to turn a term that once signified objective exigencies into a mere marker of subjective want – a tendency echoed, as we saw, by neoclassical economics – is unhelpful, stripping the concept of need of its critical leverage. If “needs of the mind” are to point to objective standards that our conceptual scheme is answerable to, we must resist Diderot’s absorption of them into the realm of the subjective.

However, we cannot simply revert to an ancient conception of cosmic necessity either. We require a conception of need that occupies the space between *Bedarf* and *Bedürfnis*: one that recognises needs as historically conditioned (arising from our specific concerns) yet structurally objective (dictated by our actual capacities and circumstances).

The rest of this paper aims to carve out this intermediate notion. I contend that conceptual needs can be objective requirements without being timeless absolutes, and that they can be something possessed unwittingly and unwillingly while being historically conditioned and reflective of individual concerns. The key to stabilising this position lies in recognising that the normativity of conceptual needs reflects neither the necessities of old nor the subjectivised goodness of the moderns, but a distinctively *aptic* normativity.

## The aptic normativity of conceptual needs

It is tempting to think that needs always imply *necessity*: that to identify a need must be to register a lack of something *necessary*. And it is true that needs-talk frequently occurs in modal locutions – both *alethic* modal locutions, such as “Oxygen is needed for combustion,” and *deontic* modal locutions, such as “You need to return the money.” In each case, the need in question indeed registers how something is necessary or required – *causally* necessary or required in the first example, and *morally* (or perhaps *prudentially*) necessary or required in the second.

This line of thought suggests that to ascribe a conceptual need is to register the lack of a concept that is necessary. The question then is: necessary *in what way*? Are we dealing with the alethic necessity that contrasts with possibility and impossibility, or with the deontic necessity that contrasts with the permitted and prohibited?

Placing talk of conceptual needs under the rubric of alethic modal locutions seems uninviting, because ascribing a conceptual need clearly registers a form of *normativity*. When we say that we need new concepts to make sense of the legitimacy of international institutions (Cueni, 2020), for example, or that we need to adapt our cognitive concepts to do justice to the emergent capabilities of AI models (Hopster and Löhr 2023), we are not making a *causal* claim, of a kind with the observation that a matchstick cannot catch fire unless we let in some air. We are making a *normative* claim bearing on our reasons for action.

This suggests that talk of conceptual needs should be placed under the rubric of deontic modal locutions instead. Yet this is not plausible either. A conceptual need is not a deontic requirement. To say that we need the concept of sexism is not to say that we are *required* to have it, as if failing to possess it constituted a violation of a duty. It is rarely, if ever, the case that we are *required* to possess a specific concept.^9^ To lack a concept is to suffer a privation, not to commit a transgression. Conversely, to say a concept is needed is to say more than that it is *permissible* to have it. This would be too weak to capture the urgency and the sense of a genuine lack that the language of “need” conveys.

My proposal, therefore, is to place conceptual needs under the rubric that Selim Berker (2022, 2024) has called the *aptic*: the distinct domain of normativity pertaining to fittingness, which contrasts with *deontic* normativity on the one hand and with *evaluative* normativity – meaning specifically the normativity of goodness or badness – on the other (note that on Berker’s account, the distinction between aptic, deontic, and evaluative normativity is orthogonal to the distinction between the various “flavours” (Berker, 2022, p. 24) of normativity, i.e. moral, legal, epistemic, prudential, etc.). When we say that we *need* a concept, we are making an aptic judgement – a judgement pertaining to the *fittingness* of the concept. A conceptual need is not a necessity in the deontic sense. Rather, it is the judgement that a certain concept is *called for* by the situation. The concept is the one that fits the situation much as a key fits a lock. Understanding conceptual needs as aptic allows us to see how they can be normatively guiding without collapsing into the unhelpfully strong notion of deontic necessity. The normativity of conceptual needs is aptic rather than deontic.

Do conceptual needs still imply necessity if they are taken to be aptic? At first pass, one might think that they must then imply some form of instrumental necessity: my needing X to achieve outcome *O* entails that *X* is necessary to achieving *O*.

But if this were so, it would severely limit the normative guidance that conceptual needs can provide for reflection on which concepts to use. For it is surely not true that the only concepts we have reason to use are those which it is *necessary* for us to use (to achieve certain outcomes). There are many concepts we have reason to use simply because they are *helpful* or *useful* in achieving certain outcomes, even though they are not strictly necessary to achieving those outcomes.^10^

Yet I think that conceptual needs can be disentangled from this implication of instrumental necessity; and the key to doing so is to recognise their aptic logic. Conceptual needs are a species of instrumental needs in David Wiggins’s (2002, § 6) sense. But when we talk about the tools we need, we are talking about *optimality* rather than necessity. In the sentence: “I used a knife to open the can, but what I really need is a can opener,” the word “need” does not signify necessity, but a judgement of *appropriateness or adequacy with respect to a task*. The speaker does not lack all means (they used a knife). But they judge their means as suboptimal or makeshift, and the can opener as functionally ideal. Thus, “I need *X*” here means something like: *“*I would achieve this goal more efficiently and effectively if I had *X*.*”* This expresses a scalar notion of instrumental fit rather than a claim about instrumental necessity.

Once this aptic logic is understood, it emerges that what we instrumentally *need* to achieve a certain outcome is rather different from what is strictly *necessary* to achieving that outcome. You *can* drive a nail into the wall using a spoon. But what you really need is a hammer. So while the outcome – the nail being driven into the wall – is achievable by other means, which entails that the hammer is not strictly *necessary* to achieving the outcome, it remains true that what the agent needs is a hammer, because a hammer would enable the agent to achieve that outcome more efficiently and effectively. The hammer is a better instrumental fit than the spoon.

This suggests that we should not treat sentences of the form:

1. *A needs X* to achieve outcome *O.*

as logically equivalent to:

(2) *X* is *necessary* to achieving *O*.

Nor should we interpret sentences of type (1) as logically equivalent to:

(3) It is *necessary*, if *A* is to achieve *O*, that *A* have *X*.

Instead, to a first approximation, we should interpret sentences of type (1) as equivalent to:

(4) For *A* to achieve *O*, it is *optimal* that *A* use *X*.

Notice that this remains a scalar notion of instrumental fit. To say that the hammer is optimal is not to say that *nothing but* the hammer meets my need. It is to say that the hammer meets my need *better* than the spoon, and even better than a heavy screwdriver.

This comes out in the way we associate needs-talk with questions of *suitability*. We commonly talk of what *suits* our needs and understand this to mean that *X* might suit them better than *Y* without implying that *Y* is altogether unsuitable. Like the notions of usefulness or helpfulness, the notion of needfulness admits of gradation (something can be *more* or *less* useful, helpful, or needful). It is important, however, not to confuse this gradation with an evaluative ranking along a single scale of goodness. The gradation is one of how closely a tool’s profile matches the specific contours of a task – more like the degree to which a key’s ridges approximate the pins of a particular lock than like a score on a general-purpose index of quality.

This is why needfulness does more to characterise the underlying scale of gradation than usefulness or helpfulness do: it organises the space of possible means not along a generic dimension of value, but as gravitating towards a specific optimum defined by the contours of the predicament. To say: “This is really useful,” or “This is really helpful,” is to say nothing about whether other things might be *more* useful or helpful, and in which direction these might be sought. To say: “This is what I really need,” by contrast, specifies the ideal fit in relation to which the fittingness or suitability of other things can then be judged.

At the same time, what counts as optimal is constrained by several factors. On the one hand, what is optimal for *A* depends on what *A*’s capacities and corresponding limitations are. The tennis racket that is optimal for Roger Federer is not optimal for me, because I lack the technical prowess to wield such a heavy, stiff, and unforgivingly small racket effectively. Instrumental needs are indexed to agents and their capacities.

On the other hand, what is optimal for *A* depends on what is realistically available to *A*. When Shakespeare has Richard III exclaim: “A horse! A horse! My kingdom for a horse!” after losing his mount at the Battle of Bosworth Field (1485) and being forced to fight on foot, it would be odd to disagree on the grounds that what Richard III really needed was a tank. As Wiggins (2002, 12) has pointed out, one’s needs are sensitive to what means are *realistically available* to one under the circumstances. How restrictive this realism constraint is taken to be will depend on the context and purpose of the need-ascription – in the context of philosophical reflection on what concepts we need going forward, we might have reason to include conceptual means that are not yet available, but that we could plausibly envisage *making* available in the near future (tanks were not among the things that Richard III could plausibly *envisage* making available). But the basic point is that instrumental optimality does not refer to some ideal technology in the distant future. It refers to what would be optimal *under the circumstances*. Needs-talk would be pointless if all it ever did was to gesture vaguely towards the ideal endpoint of human ingenuity.

Factoring in these constraints by one’s capacities and circumstances, we finally arrive at a reading of (1) on which “*A* needs *X* to achieve outcome *O*” is logically equivalent to:

(5) For *A* to achieve *O*, it is optimal, given the constraints imposed by *A*’s capacities and circumstances, that *A* use *X*.

This aptic interpretation distinguishes conceptual needs from mere evaluative claims that a concept would be “good to have.” As Berker argues, the evaluative and the aptic possess distinct logical structures. First, goodness has a *polar* opposite (badness), whereas fittingness only has a *privative* opposite (unfittingness). To be “bad” is to be “anti-good” – a substantive negative force – whereas to be “unfitting” is merely to *lack* fit (Berker, 2022, p. 46).

Second, the evaluative admits of a *neutral* state: many things are neither good nor bad, but indifferent. Conceptual needs, however, behave like fittingness in lacking this middle ground; a concept either fits a specific predicament or it does not. There is no structural state of “neither fitting nor unfitting.”

Therefore, having a conceptual need is a genuine privation: it is the privative opposite of having an apt concept. To ascribe a conceptual need to someone is to register a lack of fit between their concepts and their situation. This creates a tension that calls for resolution. Its resolution is not a contrary state – an “anti-need” – but simply the state in which the need is filled by an apt concept. The resolution of a need is thus not a movement from “bad” to “good” (like moving from pain to pleasure), but a movement from empty to filled – like a key filling a lock. This structural “lock-and-key” logic explains why judgements of needfulness can no more be reduced to evaluative judgements of goodness than to deontic judgements of requiredness. The aptic is its own kind of normativity.

Another key feature of aptic judgements is that they are not dependent on what alternatives are available. Whether a concept fits a situation depends on the features of that concept and that situation, not on a comparative ranking of all possible alternative concepts. In Berker’s guiding metaphor: “whether the key fits the lock is determined by the relation between the lock and the key, not the relation between the lock, the key, and the other keys that could possibly be used instead” (2022, 44).

## A need-first approach

If conceptual needs possess a distinctively aptic normativity, as I have argued, they provide a standard by which to judge the fittingness of our concepts. But how do we best operationalise this insight? In this section, I argue that the aptic normativity of conceptual needs comes into its own when facing problems of conceptual adaptation: situations where inherited ways of thinking no longer fit our needs and our conceptual repertoire must adapt if it is to keep step with our conceptual needs.

If our conceptual needs are to serve as a compass for conceptual adaptation, we require a method for discerning them. The challenge is formidable. The aggregate of our conceptual needs forms an intractably dense thicket of interacting forces. We cannot simply appraise our concepts by holding them up against the totality of our needs. We must find a way to render our situation intellectually tractable by disaggregating the thicket of interacting needs.

A first step in that direction is to recognise that a conceptual need is always the product of three types of factors that are need-generating when combined: *concerns*, *capacities*, and *circumstances*. This tripartite structure is what allows the need-first approach to bridge the historical divide between subjective wants and objective requirements:

1. **Concerns (the subjective pole)**: Not merely the goals we consciously pursue, but the entire range of what we care about – from our basic physiological and psychological needs to our motivations, desires, and aspirations as well as our loyalties, attachments, and commitments to particular values or projects.
2. **Capacities (the mediating factor)**: The physiological, cognitive, and technological abilities (and corresponding limitations) we can draw on upstream of adopting a concept.
3. **Circumstances (the objective pole)**: The natural and social environment we must navigate, including its threats and resources, its geographic characteristics, and its socioeconomic structures and institutions.

A conceptual need is not generated by any one of these elements in isolation. It arises from their conjunction. Note also that while the subject of these three elements could be an *individual* (when we are interested specifically in an individual’s rationale for adopting a concept already in circulation), it will normally be a *group* or *community* whose concerns, capacities, and circumstances elucidate the *initial* adoption of a concept in that group or community.

From the perspective of the individual agent, however, it can seem as though it is simply the circumstances that generate a need – the situation itself seems to call for a certain concept. This is what gives a conceptual need its air of objective exigency; it is a fitting response to a problem that the world itself has set for us. Even then, however, this problem to which a certain way of thinking is a fitting response is really the product of the interaction between what we care about, what we can do, and what the world is like. It is only through the influence of human concerns that a *mere* situation – a situation as described in the indifferent vocabulary of physics – becomes a *predicament*: a situation as described in thicker, value-laden terms expressing a practical tension calling for resolution. That is why, to appreciate how we find ourselves in predicaments calling for certain concepts, we need to analyse the relevant situations not just in terms of circumstances, but also in terms of the concerns and capacities we bring to them.

To see the merit of this tripartite analysis, consider three cases where a change in any one of these three variables – first circumstances, then capacities, and then concerns – can generate or obviate a conceptual need.

First, consider the concept of *commute*. For there to be a conceptual need for such a notion, three factors must come together: (1) a concern to secure one’s livelihood by regularly discharging work obligations that require physical presence at a fixed workplace; (2) the capacity for regular travel; and, crucially, (3) the *circumstances* of an industrialised society in which home and workplace are spatially separated. A medieval peasant, living on the land he tilled, lacked the relevant circumstances and therefore lacked the conceptual need characteristic of industrialised societies with clearer separations between home and workplace. But change the circumstances yet again, as widespread remote work technology is now doing, and for many, that spatial separation dissolves once more. These new circumstances increasingly obviate the need for the concept of a commute, while generating a need for new concepts – such as *work-life integration* – to make sense of the professional sphere collapsing back into the domestic.

Second, consider the concept of *close contact* that emerged as part of the contact tracing efforts during the COVID-19 pandemic. The concern (to protect public health) as well as the circumstance (a pathogen spreading rapidly) were present before, and fourteenth-century port authorities in Ragusa and Venice already imposed quarantines on ships coming from plague-infested areas (Mackowiak & Sehdev, 2002). But the specific conceptual need for an exactly defined notion of *close contact* (e.g. being within six feet for at least fifteen minutes) only arose because we developed particular *capacities*: sophisticated epidemiological models and the digital infrastructure to precisely track the relevant parameters. Medieval authorities had no need for such conceptual precision because they lacked the capacity to act on it; a cruder concept of *proximity to the afflicted area* sufficed.

Finally, consider the concept of *burnout*. Experiences of profound exhaustion from work are not new. But the concept of burnout more specifically picks out a state of exhaustion accompanied by cynical detachment from one’s work and by a sense of ineffectiveness or failure. The need for this concept arose not primarily because our capacities for work or the material conditions of labour changed, but because our *concerns* did. In a society whose dominant concerns centre on honour or sheer survival, work-related exhaustion is more readily conceptualised in terms of weakness or unfortunate necessity. The concept of burnout becomes needful only in a society that places importance on personal fulfilment and self-realisation *through* work. It is the clash between this modern concern for work to be meaningful and the reality of an unfulfilling job that gives point to the concept of burnout.

In each of these examples, it is the interaction between certain concerns, capacities, and circumstances that generates a conceptual need. A concern alone will be impotent or inert unless refracted through the right capacities and circumstances. Even if we analytically distinguish these three components of a situation, however, we still cannot comprehensively consider all the relevant concerns, capacities, and circumstances at once.

The solution, I suggest, is to approach this complexity piecemeal, by constructing what I have called a *need matrix* (2025, 232): an interpretative model or schema that isolates a minimal set of conditions jointly sufficient to generate a specific conceptual need. Bernard Williams (2001, 92) used the term “matrix” in roughly this sense, presumably drawing on the term’s etymological evocation of the womb or the mould doing the work of generating and shaping something. Fittingly for our purposes, the term “matrix” is commonly used to refer both to the environment out of which *ideas* develop and to the “printer’s matrix” – the mould from which printing type is cast.

Like a printer’s matrix, a need matrix can act as a mould for fitting concepts. I thus use the term primarily in its etymological sense of a *generative structure* rather than in the linear-algebraic sense of a transformation operator. A need matrix does not profess to be an exhaustive description of the forces acting on concept formation, but a perspicuous representation of a single, powerful dynamic that concept formation should be responsive to. Such a perspicuous representation can then be critically evaluated and perhaps complemented or counterbalanced by further need matrices, thereby building towards a good approximation of the landscape of our conceptual needs.

Constructing a need matrix is a delicate interpretative task. We can do so *retrospectively*, by reconstructing what need matrix, if any, underpins one of our existing concepts; or *prospectively*, by constructing a need matrix we believe captures an important aspect of our predicament and using the matrix as a guide to what concept we need.

If starting from an existing concept, a useful heuristic is to treat two of the three variables in the matrix as constants and solve for the third. Given some concept *F*, we can, for instance, treat some of our actual capacities and circumstances as given and ask: what concern would engender a need for a concept like *F* when pursued with these capacities under these circumstances? This narrows the search space and provides a criterion for what counts as a solution. By identifying a concern that renders *F* needful for agents like us in circumstances like ours, we uncover a reason for its use.^11^

When applied to concepts we do not yet have, on the other hand, the same approach becomes a guide to conceptual adaptation. Instead of starting from an existing concept, we can begin with a discerned predicament – a thickly described situation of practical tension which makes our current conceptual repertoire seem lacking. We then construct a need matrix that models this predicament. This matrix functions as a blueprint, or a mould from which to cast a fitting new concept. It moves us beyond simply polishing away inherent flaws such as vagueness, open texture, or inconsistency, and instead presents the task of ameliorating our conceptual resources as one of situated adaptation.

Since the concept itself is the variable we are solving for when the aim is to use a need matrix as a blueprint for a concept we do not yet possess, the challenge is to arrive at an accurate and helpful understanding of our own predicament, because all three components of the need matrix will have to be in place in order to yield a determinate blueprint. Guiding questions in constructing a need matrix include the following: do the conditions specified in the matrix actually obtain? And do we, upon reflection, identify with the concerns represented in the matrix?

Indeed, one strategy for arriving at a plausible and interesting need matrix is to combine the retrospective and the prospective: it can be helpful to *start* by reconstructing the need matrix behind an existing concept before considering how this need matrix may have been altered by recent disruptions. In other words, we chain together the retrospective and prospective uses of a need matrix. This acknowledges the point, made by several theorists of conceptual engineering, that it is a good idea to reverse-engineer what our existing concepts do for us before we tamper with them, and that this reverse-engineering can guide our engineering.^12^ Indeed, this two-step matrix construction is how Hopster and Löhr’s injunction to adapt concepts by preserving their function in the face of new circumstances is best accommodated within the need-first approach. The subtle difference, however, is that considering how a retrospectively reconstructed need matrix is being reconfigured by social or technological disruption may lead one to the conclusion that the concept we now need should serve a *new* function.

Once such a need matrix is in place, it can tell us what a new or revised concept needs to *do*. It can help specify the desiderata for the needed concept: what it needs to track in the world, what inferential consequences should follow from its application, and what practical orientation it should provide.

Perhaps a helpful way to visualise the guidance provided by such a need matrix is to view the interplay between these parameters as resulting in a specific *need vector*. Just as distinct physical forces acting on a body combine via vector addition to produce a single resultant force, so too the pressures acting on our conceptual repertoire – the weight of our concerns, the possibilities and constraints of our capacities, and the push and pull of our circumstances – combine to determine a specific direction in conceptual space. In this geometric picture – offered here for purposes of intuitive visualisation rather than precise formalisation – the need matrix represents a collection of component forces or vectors, while the need vector represents their summation: it is the *resultant* of the component vectors represented in the need matrix. To say that a certain concept is the one we really need is to say that, of the realistically available options, it is the one that most closely aligns with that resultant trajectory.

Such a need-first approach allows for a multi-faceted appraisal of existing concepts. In light of a need matrix modelling the conditions generating a need for a certain concept, that concept might be revealed to be unsatisfactory because the need it once met has vanished with changing capacities and circumstances; or because we no longer share the concern that animated its use; or simply because, while the need is real, the concept is a clumsy or ill-fitting tool for the job. The need-first approach thus invites us to ask three critical questions once a plausible need matrix for a concept is proposed:

- **Matrix Applicability**: Do the conditions specified by the matrix actually obtain? Are these concerns, capacities, and circumstances in fact conjoined as the need matrix suggests?
- **Aptic Fit**: How well does the concept under scrutiny meet the resulting need? Is it an apt tool?
- **Normative Endorsement**: Do we, upon reflection, endorse the concern that the matrix identifies as the source of the need? Is this a concern we want to see satisfied?

One has a *pro tanto* reason to use a concept only if one can give an affirmative answer to all three questions.

To illustrate how a reconfigured need matrix can reveal the need for a new function, consider the rapid obsolescence of our concept of *privacy*. For much of the modern era, our operative concept of privacy was the traditional, liberal conception of *privacy as seclusion*. On this conception, privacy is fundamentally a shield against intrusion. It was first given its canonical legal expression as a “right to be left alone” by Samuel Warren and Louis Brandeis in “The Right to Privacy” (1890). Their argument was a direct response to a shift in the technological capacities and circumstances of their time: the advent of instantaneous photography and the rise of a sensationalist press with the power to disseminate personal images and gossip to a mass audience. Their proposed concept was thus a tool perfectly tailored to the need they diagnosed: a shield apt for a world where intrusion was a physical, targeted act.

But the rise of big data and artificial intelligence means that this conception of privacy no longer fits the world we live in. As Shoshana Zuboff (2019) has documented in her analysis of “surveillance capitalism,” the threat is no longer just an unwanted intrusion into a private space, but the pervasive harvesting and analysis of the data trails we voluntarily leave behind. The danger is less that someone will read one’s diary, and more that an algorithm will, with frightening accuracy, *infer* the contents of a diary one never wrote. In a world in which aggregating thousands of data points – online searches, location history, typing cadence – makes it possible to predict anything from people’s personality through their vulnerabilities to their future behaviour, the chief threat is not intrusion, but inference. When the relevant data are voluntarily surrendered in exchange for a service, this does not qualify as a violation of privacy as seclusion. Yet it constitutes a sufficiently serious threat to the animating concern behind the concept of privacy – the concern to preserve our dignity and autonomy – to warrant a revision of the way we conceive of privacy.

In fact, if we hold these novel technological capacities and circumstances constant and ask what concern might animate the use of the concept of privacy under these conditions, we realise that these new powers and threats affect the character of the underlying *concern* as well. The concern can no longer simply be one for dignity and autonomy through non-observation. As Zuboff argues, it must become one for dignity and autonomy *through informational self-determination*. This is not just the concern to be left alone, but to control the flow of one’s personal information – to know what is being collected, and to be able to correct it and contest its use. This concern is not merely personal but, as Carissa Véliz insists, fundamentally political. In *Privacy is Power* (2020), she argues that we are ceding power by ceding data, creating structural asymmetries that threaten the foundations of democratic autonomy by empowering those who control our data to predict, pre-empt, and modify our choices.^13^

The conceptual need that arises from this change in our predicament is for a new conception of privacy – one that is not about hiding information but about controlling its inferential potential. It is a need for a concept that can make sense of the harm done by prediction rather than disclosure, and that can ground a right not to be rendered transparent and predictable by an algorithmic gaze .

We can capture the structural depth of this obsolescence by placing the need matrix for the traditional concept side-by-side with the matrix for the predicament we now face (see Table 1).

**Table 1:** The shift in conceptual needs underpinning the concept of privacy

	Matrix 1: Privacy as Seclusion (c. 1890–1990)	Matrix 2: Privacy as Self-Determination (Present)
Concerns	To maintain personal dignity and autonomy by protecting personal life from intrusion.	To maintain personal dignity and autonomy by controlling the inferential potential of one’s data trail.
Capacities	Photography and mass media enable the documentation and dissemination of personal life, but this required physical and targeted intrusion (paparazzi, wiretaps).	Machine learning and ubiquitous computing enable the aggregation, cross-referencing, and analysis of voluntarily surrendered data to create powerful predictive models.
Circumstances	Data were analog, ephemeral, and scattered.	Data are digital, persistent, and centralised.

When mapped against these matrices, the normative status of our concepts becomes clear. The concept of *privacy as seclusion* was a fitting response to Matrix 1. In relation to Matrix 2, however, that same concept is no longer fitting. It fails to register the relevant threat because that threat is no longer intrusion, but inference.

By contrast, a concept of *privacy as informational self-determination* aligns with the new need vector generated by Matrix 2:^14^ it tracks the right threat (inference rather than intrusion), enables the right inferences on the part of those who wield it (about who controls data flows and to what end), and orients action toward the right response (institutional constraints on data aggregation rather than thicker walls around the domestic sphere). It thereby answers to the specific concern for control over prediction that arises when new, AI-enhanced capacities for inference are conjoined with the circumstances of surveillance capitalism.

This illustrates how the aptic normativity of conceptual needs can guide conceptual adaptation. Once the shift in our conceptual needs is made perspicuous through the representational device of need matrices, the demand for conceptual adaptation appears not as the aspiration to sharpen or smoothen our concepts, but as a call to realign our thought with our reality. Clinging to the old concept would be a way of systematically mischaracterising, and hence mishandling, the predicament we are in.

Conceptual engineering, on this picture, is not an exercise in polishing away the inherent flaws of concepts in the isolation of the philosopher’s lens-grinding workshop; it is an expression of the age-old aspiration to *adaequatio intellectus ad rem*: the adequation of the intellect to things – interpreted, however, as the practice of continually tailoring our conceptual repertoire to the aptic pressures exerted by our evolving concerns, capacities, and circumstances.

## Needs, goals, and functions

One might wonder, however, whether those “needs of the mind” are ultimately reducible to the goals we pursue or to the functions our concepts serve. To demonstrate the distinctiveness and independence of the need-first approach, we must contrast it directly with its two closest rivals: the approaches that appraise concepts by the goals of concept-users or by the functions of concepts.

### Needs vs. goals

The idea that we should assess concepts by starting from the goals, aims, or purposes we pursue with them has proven popular in the literature on conceptual engineering (Brigandt 2010, 2011, 2012; Burgess and Plunkett 2013a, b; Brigandt and Rosario 2020; Pérez Carballo, 2020; Nado, 2021). As Ingo Brigandt and Esther Rosario have recently argued, for example,

there are cases where a scientific aim can be tied to an individual concept in that this concept is being used by scientists to pursue this aim. For example, while the classical gene concept was used for the purpose of predicting (and statistically explaining) phenotypic patterns of inheritance across generations, the molecular gene concept serves the aim of causal-mechanistically explaining how inside a cell a gene leads to the formation of its molecular product. Making explicit such an aim tied to a concept’s use permits one to account philosophically for the rationality of concept change: a revised definition is an improvement over an earlier definition if the former is empirically more conducive to meeting this aim. (Brigandt and Rosario 2020, 102)

The idea here is “to view a concept as being used by scientists to pursue a *specific scientific aim*” (Brigandt and Rosario 2020, 102). Presumably, however, there will also be many cases where it is less straightforward to tie an individual concept to a specific aim (whether scientific or not). Unlike function-first approaches, which invite one to focus on *the* function of a concept, goal-first approaches must reckon with the fact that concepts are used to pursue all sorts of aims. But some, like Jennifer Nado (2021), see this as an advantage. They regard talk of “goals,” “aims,” or “purposes” as superior to talk of “functions” precisely because, while some have denied that concepts *have* functions in any interesting sense (Cappelen, 2018), it is plainly apparent that “humans certainly have purposes and goals and aims; and it’s not particularly problematic to claim that we often use concepts to help us achieve those purposes, goals, and aims” (Nado, 2021, S1521).

The basic idea has undeniable attraction: a revised concept is better iff it allows us to achieve our goals more effectively. This standard is also straightforwardly epistemically accessible: we generally know what we intend to achieve. Of course, goals vary from person to person, and people continually deliberate about which goals to adopt or discard. This means, as Amie Thomasson acknowledges, that “there are deeper questions one can raise about which goals we should adopt” (2020, 440). But she proposes to work around this by “*presupposing various widely shared goals that are generally presupposed in debates about what concepts to use*” (2020, 440).^15^ A goal-first approach might then start by “keeping fixed some goals we wish to fulfill or see as desirable” and asking how our concepts can “help fulfill these goals” (2020, 440).

Yet making concepts answerable to a standard that is so epistemically accessible and responsive to our will can also become a liability by rendering the approach too *intellectualist*. If we presuppose that we already know what we are aiming for with our use of a given concept, we will be ill-placed to recognise how concepts often serve us in ways we do not consciously intend.

To develop this point, it is worth following Steffen Koch and Christian Nimtz (2025) in distinguishing more clearly between “goals” (*Ziele*) and “concerns” (*Anliegen*). If by “goals” we mean the immediate aims we pursue with our actions, then “concerns” stand at one remove from goals: they are the things we ultimately care about that animate the wider practice of which these particular actions are instances. We consciously pursue our immediate goals *with a view to* realising certain concerns. While our goals are the explicit aims with which we do things, and hence something we are necessarily conscious of, concerns are part of the evaluative background against which we act; though they implicitly animate our practice, they are not necessarily something we are conscious of as we act; we may not even understand how the practice that our action instantiates relates to our concerns. Though those concerns rationalise the practice, the rationales themselves (“We have reason to engage in practice *P* because it serves our concern for *X*”) might not be represented by anyone. They might, in Daniel Dennett’s phrase, be “free-floating rationales” (2012).

The contrast becomes even sharper if we distinguish *particular uses* of a concept from the *practice of using* that concept. A particular use of a concept might serve an immediate goal; but the practice of using a concept typically does not serve an immediate goal, even though it typically does serve *some* human concern (unless it is a purely vestigial practice, now detached from every human concern). To see the force of this distinction, consider the legal concept of due process. A defence attorney might use this concept with the immediate goal of suppressing evidence. But what gives the *practice* of using the concept of due process its point is not that immediate goal, but rather that the concept serves the deeper human concerns for justice and the protection of individual freedom against the power of the state. The attorney may or may not be aware of these underlying rationales; in the heat of the moment, certainly, her attention is likely to be absorbed by the immediate tactical goal she is pursuing in using the concept. And yet it is those background concerns for justice and freedom that provide the ultimate rationales for making this goal-directed appeal to due process conceptually available within legal proceedings.

This distinction is crucial for conceptual adaptation. It is those underlying concerns and the conceptual needs they generate, rather than the immediate goals of concept-users, that provide the ultimate standard by which we can appraise the concept of due process. In fact, in this particular example, engineering the concept of due process to maximise its conduciveness to satisfying the attorney’s immediate goal (suppressing evidence) would even *undermine* the concerns animating the practice of using the concept (justice and freedom).

Appraising concepts by goals alone thus risks missing the forest for the trees. A great many of our conceptual tools meet needs we are not aware of, because they are not consciously pursued as goals. As Millikan’s reflection on sexism brought out, we can have a need for a concept long before we have the goal *of* deploying it or any goals *in* deploying it. Conceptual needs might be possessed *unwittingly*. Unlike our goals, they can come as a discovery.

Indeed, one might think that it must be that very discovery, however inchoate, that then motivates the conscious adoption of the goal of introducing the concept. A group suffering what Miranda Fricker calls “hermeneutical injustice” (2007) because they lack the concept of *sexual harassment* does not yet have the goal of deploying that concept (since that would require having the concept already). What they have is a pressing, if unarticulated, conceptual need. This need exists prior to the eventual goal of introducing a new concept; and, once brought to awareness, this need is what motivates and justifies that goal.

A further difference is that goals are not just something we consciously adopt, but also something we can simply discard, as Jennifer Nado puts it (2023, 1986). Conceptual needs, by contrast, are typically neither something we consciously adopt nor something we can discard at will. They might be possessed not just unwittingly, but *unwillingly*. Even insofar as our conceptual needs depend on what our concerns are, these are in important respects not subject to our will in the way our conscious goals are – there are some things we cannot help but be concerned with, and we may be concerned with them even if we do not consciously pursue them as goals.

A need-first approach is thus better placed to acknowledge that the deepest reasons we have to use certain concepts are often grounded in something less responsive to our will than what goals we decide to adopt or discard. Goal-first approaches threaten to become not only too intellectualist, but also too *voluntaristic*.

In sum, because needs are rooted not just in our goals, but in our underlying concerns, and not just in our concerns, but also in our capacities and objective circumstances, they provide a less intellectualist and less voluntaristic standard of appraisal. They are closer to the notion of *Bedarf* – objective exigencies imposed on us by our predicament – than to the notion of *Bedürfnis* – felt lacks arising from the subjective goals we have adopted and could easily discard.

### Needs vs. functions

In search of a more objective alternative, many look to the functions of concepts (Prinzing, 2018; Haslanger, 2020; Simion and Kelp 2020; Riggs 2021; Jorem 2022; Queloz 2022a; Thomasson 2022; Köhler and Veluwenkamp 2024; Thomasson, 2025; Zuber, 2025). Hopster and Löhr’s (2023) account of conceptual adaptation also belongs in this family: they suggest that, under changing circumstances, we ought to preserve the inherited function of our concepts as far as possible, adjusting their application and extension so that they can continue to do what they have been doing for us all along. By contrast, the need-first approach asks not how to safeguard a concept’s established function in new conditions, but how to secure apt conceptual responses to the predicaments we actually face. This makes conceptual adaptation a matter of adjusting our conceptual repertoire to whatever we now need it to do for us.

This need-first framing has three advantages over the function-first framing. One is that the notion of a conceptual need is inherently *agent-centred*: a conceptual need is always *someone*’s need, whether an individual or a group. Needs-talk thus always raises, and keeps in view, the question of *whose* needs are in question. This builds the *cui bono-*question – the traditional guiding question of ideology critique – right into the grammar of concept appraisal. Indeed, while the *cui bono* question tracks the distribution of benefits in the hope that those whose role merits critical scrutiny will be rendered salient by the accumulation of their gains, asking “*cui opus*?” – “Who needs it?” – promises to provide sharper diagnostic guidance.^16^ Benefits can sprawl and cascade in many directions, often accruing incidentally or temporarily to agents who have no structural stake in the concept. But asking who *really needs* a certain arrangement narrows down the search space more efficiently. This precision stems from the aptic nature of needs: because needs organize the space of possibilities around optimal fit, identifying a need isolates the specific “lock” that the concept fits as a “key.” We are not, therefore, merely asking who happens to benefit from the way things are; we are asking for whom this specific arrangement constitutes a structural solution to a predicament.

By contrast, talking of the functions of concepts makes it tempting to consider concepts and their functions in isolation, as if the function were a property of the concept itself. Talk of the function of a concept thus encourages a slight but important attentional shift away from the agents whose lives the concept shapes and towards the concept as an apparently autonomous bearer of functionality.

A second advantage is the built-in *pluralism* of needs-talk. We naturally think of agents as having many needs. A single concept can therefore be appraised in terms of the various respects in which it meets or fails to meet our various needs.

Function-talk, by contrast, gravitates toward the singular: it invites us to look for *the* function of a concept. We can of course explicitly acknowledge that a concept can have multiple functions, but the idiom itself implicitly nudges us to consider one function as primary.

But most importantly, conceptual needs are *prospective* where conceptual functions are retrospective. This backwards temporal orientation is built into both of the two main theories of function that have been imported into the conceptual engineering literature. On accounts drawing on *etiological* theories of function, which go back to Wright (1973), Millikan (1989), and Neander (2017), the function of a concept is whatever effect historically contributed to its continued use and transmission (Simion, 2019; Thomasson, 2020, 444). On accounts drawing on *causal-role* theories of function, which go back to Cummins (1975), a concept’s function is the contribution it currently makes to some larger system (Haslanger, 2020; Thomasson, 2022, 2025).

Both theories of conceptual functions are in crucial respects backward-looking, rendering function-first engineering prone to over-indexing on past functions. Etiological accounts identify functions by reference to selection histories, making them inherently historical; causal-role accounts identify functions by reference to causal contributions to an already up-and-running system, which is still a reflection of the recent past and makes those accounts, if not backwards-looking, then at best sideways-looking. Both kinds of accounts are well suited to answering the question: “What has this concept been doing for us?” But they are imperfect guides when the terrain shifts and we need to know what a concept *ought* to become.

The case of *privacy* illustrates this limitation. On both etiological and causal-role accounts, the function of the traditional concept of privacy was to act as a shield against intrusion. It persisted because it successfully protected the domestic sphere from intrusion. If we followed a function-preservationist approach, we would try to adapt the concept to continue serving this function of shielding against intrusion.^17^

But, as the analysis in § 4 showed, this is precisely what we do not need. In an age of big data and AI-powered inference, a shield against intrusion is rather like the Maginot Line – functionally intact, but strategically inept. We need a new function: control over the inferential potential of data trails. A need-first approach is nimble enough to discard the old function because it is guided by the current predicament rather than by the history of the tool. Needs-guided conceptual adaptation goes by what we now need our concepts to do for us, not by what they have functioned to do in the past. Prospectivity is built into the very starting point: to identify a conceptual need is to register a lack – a gap between our current conceptual repertoire and what our evolving concerns, capacities, and circumstances call for. A need-first approach is therefore particularly well suited to the challenge of conceptual adaptation in unprecedented circumstances, where guidance must come from an assessment of what would be fitting now and going forward, not from what has been functional thus far.

Recognising this problem, some theorists have proposed forward-looking conceptions of function. In “Conceptual Innovation, Function First” (2020), Mona Simion and Christoph Kelp introduce the notion of a *design-function* (“d-function”) alongside the notion of an etiological function (“e-function”). A thing has a d-function in virtue of what its *designer intends it to do*. On their picture, conceptual engineers are, “in the first instance, designers” (989): they either assign new d-functions to existing concepts or introduce new concepts with new d-functions. A conceptual engineering project then counts as successful if the engineered concept spreads and persists *because* it fulfils that d-function reliably enough – that is, when its d-function is converted into an e-function.

This is an elegant picture, but it is normatively much closer to a goal-first approach than the “function-first” label suggests. Since d-functions are fixed by the intentions of designers – what they mean their concept to do for us – the normative standard of appraisal for the concept is ultimately set by those intentions. The fact that a concept’s continued use would, if all goes well, also imbue it with an e-function does not change this; it simply adds a retrospective validation of the designers’ aims. As a standard for appraisal, however, d-functions face the same problems as goal-first views: they go by the conscious and willingly adopted aims of those who design or introduce concepts rather than by the largely unappreciated conceptual needs that people have *nolens volens*.

In addition, one might worry that the agents most likely to get to choose d-functions (theorists, legislators, and corporations) are not the ones whose conceptual needs are most pressing; yet a conceptual innovation would count as a success on Simion and Kelp’s account – it does what its introducers meant it to do and gains a foothold in our practices for that reason – even if, relative to the conceptual needs of those living by the concept, it is a positively ill-fitting or oppressive tool. As an account of normativity, d-functions thus inherit the strengths and weaknesses of goal-first views rather than offering a genuine alternative.

Another attempt to devise a forward-looking notion of function is Queloz’s (2022a) concern-satisfaction account. On this view, the function of a concept is given by the way its use tends, under propitious circumstances, to contribute to the satisfaction of concept-users’ concerns. This is not a matter of anyone’s having represented this contribution as a goal; it is enough that the concept, when properly deployed, typically makes a useful difference to the realisation of people’s concerns. This account is prospective because a concept’s possessing such a *concern-relative function* (“c-function”) is a matter of there being an instrumental relation between a concept and a concern, which is the case not just when the concern is actually being satisfied by a concept’s application, but also when the concern *would* be satisfied if the concept *were* to be applied. A concept might thus possess a c-function even if that c-function has not yet been performed.

However, Aneta Zuber (2025) has raised difficulties for this account. It struggles to differentiate true functions from accidentally useful effects, she argues, and it risks circularity: absent an independent account of what makes circumstances “propitious,” the appeal to propitious circumstances threatens to become a tautology (circumstances are propitious iff the function is performed). Moreover, Zuber worries that because the account focuses on a concept’s positive contribution to the satisfaction of a concern, it has an optimistic and hence overly conservative bent, threatening to relegate the harmful aspects of a concept’s operation to the status of mere “side-effects” of a basically beneficial function.^18^

The need-first approach I advocate here offers a way to retain the prospective spirit of this account while avoiding its pitfalls. It does so by replacing the vague appeal to “propitious circumstances” with the explicit articulation of a need matrix.

Articulating a need matrix solves the circularity problem. We do not need to appeal to an unspecified set of “propitious circumstances.” The matrix is the specification of the relevant circumstances (along with concerns and capacities). We can straightforwardly ask: does the concept fit this matrix?

Second, the matrix solves the problem of accidental effects. Centrality is indexed to the matrix. An effect is “functional” – or rather, meets a need – if it aligns with the need vector generated by the specific interaction of concerns, capacities, and circumstances. Other beneficial effects are, from the perspective provided by that need matrix, incidental.

Finally, a need-first approach does not relegate harmful operations to the status of mere side-effects. Precisely because needs are always *someone*’s needs, such an approach can leverage the question of who benefits *in the service of critique* exactly as the traditional “*cui bono*?”-question of ideology critique does. The construction of a matrix holds up to critical scrutiny the concern that figures as the source of the need, and a key question raised thereby is whether the concern is one we endorse. A concept may be exquisitely apt relative to a matrix built around a concern for exploitation, or for racial purity. In such cases, uncovering the relevant need matrix can itself be an *indictment* of the concept, because it reveals that its very aptness is indexed to a sinister standpoint. Here, the need-first account does precisely what Zuber demands: it does not relegate the problematic effects of the concept to the status of mere “side-effects,” but locates the morally troubling concern at the core of the concept’s rationale. It is worth recalling that it was Nietzsche who made it a guiding principle that “our *concepts* are inspired by our *need*” (2009, 1885, 2[77]), and he was interested in conceptual needs primarily for critical purposes: he suspected that they would expose many concepts as apt only from the perspective of highly unsavoury characters.^19^ There is no presumption that only virtuous minds have needs.

In this way, the need-first approach accommodates much of what is best in function-first views while avoiding many of their shortcomings. This is not to say that needs-talk is necessarily incompatible with function-talk: one might define functions in terms of needs (Queloz, 2021, 221–27).^20^ The point is rather that function-talk is most illuminating when treated as derivative: as a convenient shorthand for the ways in which an apt concept would operate within a given need matrix. The deep normative work is then done, not by “the functions of concepts,” but by the conceptual needs that arise at the intersection of our concerns, capacities, and circumstances.

If conceptual needs are better suited to guide conceptual adaptation than either goal-first or function-first approaches, then, it is because, rather than extrapolating from the goals we already consciously adopted or the functions that our concepts already serve, *they direct our attention to understanding the often unprecedented predicaments we unwittingly and unwillingly face*. To seek to discover our conceptual needs instead of looking to goals or functions acknowledges the respects in which the standards governing our concepts are *objective*, *opaque*, and *dynamic*: objective, because those standards largely depend on what our capacities and circumstances actually are; opaque, because these standards may not yet be fully transparent to us, requiring more inquiry before they can fully come into awareness and inform what goals we set ourselves; and dynamic, because, as the rise of artificial intelligence reminds us, those standards are liable to shift abruptly, requiring nimbler and more radical conceptual adaptations than can be read off from established goals or functions alone.

## Conclusion

This paper has sought to recover and repurpose a fundamental normative standard: the needs of the mind. I have argued that when we seek to assess and improve our conceptual resources, our primary guide should be neither subjective goals nor historical functions, but the evolving conceptual needs arising at the intersection of our concerns, capacities, and circumstances.

My proposal rests on two connected claims. The first is that conceptual needs possess a distinctively aptic normativity. A conceptual need registers a lack of fittingness – a mismatch between our conceptual repertoire and our predicament. It is neither a deontic requirement to possess a concept nor a mere evaluative judgement that a concept would be good; rather, it signals a privation that calls for resolution. The normativity of conceptual needs is a normativity of fit – like that of a key to a lock, or of blocks of type to a printer’s matrix.

Second, to make this normativity tractable, I have introduced the device of a need matrix. By modelling a predicament as a specific conjunction of concerns, capacities, and circumstances, we can glean what I have called the “need vector” indicating the direction in which our concepts must evolve. Conceptual engineering thus becomes a problem of vector alignment: finding the concept that aligns most closely with the resultant force of our needs.

This need-first approach offers distinct advantages over its main rivals. Unlike goal-first approaches, it avoids intellectualism and voluntarism, acknowledging that our most pressing needs are often possessed unwittingly and unwillingly. And unlike function-first approaches, which remain tethered to the past, the need-first approach is inherently prospective, rendering it uniquely suited to guiding conceptual adaptation during times of social and technological disruption.

In the end, attending to the needs of the mind reorients conceptual engineering away from detached amelioration and towards situated adaptation. It calls us back to the task of *adaequatio intellectus ad rem*: not as limning the contours of a static world, but as continuously tailoring our conceptual repertoire to shifting demands. The task is to ensure that our concepts do not become vestiges of a vanished world, but remain apt for the one we inhabit.  

## Data Availability

Not applicable.

## Footnotes

1 The account offered here extends and refines rather than restates the account I gave in *The Ethics of Conceptualization: Tailoring Thought and Language to Need* (2025). Readers of that book will thus find fresh material here.

2 See Braybrooke (1987); Thomson (1987); Wiggins and Dermen (1987); Brock (1998); Wiggins (1998, 2002); Reader, (2005); Thomson (2005); Reader (2007); Scheman (2011); Simmons (2015); Brock and Miller (2019); Colton (2023); Shaw (2023).

3 Kusch (2017) analyses the *Sprachnot* (linguistic despair) of Holocaust survivors in reporting their horrendous experiences; Kusch and Ratcliffe (2018) do the same for chronic pain. On the difficulties involved in articulating a thought even in more mundane cases, see also Alshanetsky (2019).

4 Which is not to deny the importance and power of words; see Henning et al. (2025) for a particularly lucid survey of that power.

5 See Hume (2000, 3.2.2).

6 See Rousseau (1977).

7 In economics, the methodological rejection of “needs” in favor of “wants” or “preferences” traces back to Robbins (1932) and was popularised by Alchian and Allen (1983).

8 This is not to say that Ancient Greek thinkers only thought of needs in terms of objective constraints. They employed multiple terms – notably ἀνάγκη (*anankē*), δεῖ (*dei*), and χρή (*chrē*) – to capture different shades of need, including needs arising from desires; Plato, Aristotle, and Epicurus were all in different ways concerned to limit the power of needs reflecting “unnecessary desires;” see Barrett, (1964), Williams (1993), and Konstan (2025). The point is rather that in the ancient world, these subjectivised needs coexisted alongside more external, objective needs.

9 There arguably are some instances of this, however; see Smyth (manuscript) and Queloz (2024).

10 I am grateful to an anonymous reviewer for pressing this point.

11 This kind of *practical clarification* of the concepts we have already, to use Carnap’s term, can guide their optimisation for the needs they to some extent already serve. For a detailed account of how to deploy need matrices retrospectively, see Queloz (forthcoming).

12 See Plunkett (2016), Thomasson (2020, 2025Queloz (2021, 2022b).

13 Including by influencing which concepts we frame our thinking in; see Queloz and Bieber (2022).

14 See Rouvroy and Poullet (2009) for a detailed account of how informational self-determination relates to the values of dignity, self-determination, and autonomy.

15 A complication in Thomasson’s account is that she then moves from goals to the *functions* that concepts should serve to help fulfill those goals, making her account a hybrid in relation to my discussion. I address the functionalist aspect below.

16 Short for *cui opus est* – I chose *opus* over other Latin words for need (like *necesse* or *egestas*) because of its etymological roots, which align with my characterisation of need in terms of aptic normativity. *Opus* comes from the root for *work* or *task*. When one says *opus est* in Latin, this usually implies that one needs a tool or a resource to complete a specific task.

17 Thomasson (2020), cognisant of this problem, makes it part of her framework to question whether a function should be preserved.

18 See also von Samson (manuscript) for a particularly forceful articulation of this line of critique.

19 For detailed interpretations of these passages in Nietzsche, see Queloz (2017, 2023).

20 But then it is the needs that are doing the normative work, and function-talk becomes a convenient redescription of that work rather than an independent source of guidance. The present account draws this consequence explicitly, putting the issue directly in terms of needs and grounding them in the tripartite structure of concerns, capacities, and circumstances.
